# Minimally Invasive Cardiac Surgery for Spontaneous Coronary Artery Dissection

**DOI:** 10.70352/scrj.cr.26-0063

**Published:** 2026-04-28

**Authors:** Rena Usui, Wataru Uchida, Tsubasa Yazawa, Akihiko Usui, Masato Mutuga

**Affiliations:** 1 Department of Cardiovascular Surgery, Fujita Health University Okazaki Medical Center, Okazaki, Aichi, Japan; 2 Division of Cardiac Surgery, Toyota Memorial Hospital, Toyota, Aichi, Japan; 3 Department of Cardiac Surgery, Nagoya University Graduate School of Medicine, Nagoya, Aichi, Japan

**Keywords:** minimally invasive cardiac surgery coronary artery bypass grafting (MICS-CABG), spontaneous coronary artery dissection (SACD), surgery

## Abstract

**INTRODUCTION:**

Published evidence on coronary artery bypass grafting for spontaneous coronary artery dissection (SCAD) is limited, and the optimal surgical strategy for SCAD has not yet been established. We herein report a case of SCAD treated with minimally invasive cardiac surgery coronary artery bypass grafting (MICS-CABG). This is the first case of SCAD treated with MICS-CABG in the literature.

**CASE PRESENTATION:**

The patient was a 50-year-old man who was admitted to the emergency room with sudden chest pain. Emergent coronary angiography revealed double-lumen findings in the left main trunk (LMT). We diagnosed him with SCAD and decided to treat him conservatively. However, his troponin I (5549 pg/mL) and creatine kinase (533 U/L) levels increased, and coronary CTA (CCTA) revealed that the true lumen of the LMT was compressed to 75% stenosis on the next day. We performed MICS-CABG with the left internal thoracic artery (LITA) anastomosed to the left anterior descending branch via a small left thoracotomy, because the myocardial infarction was impending. The operation time was 182 min, and intraoperative blood loss was 368 mL. The postoperative course was uneventful. The SCAD lesion improved completely, and the LITA was patent on re-evaluation 3 months after surgery.

**CONCLUSIONS:**

In patients with a relatively stable hemodynamic status, MICS-CABG is a feasible and effective therapeutic approach for SCAD.

## Abbreviations

ACS
: acute coronary syndrome
AST
: aspartate aminotransferase
CABG
: coronary artery bypass grafting
CAG
: coronary angiography
CCTA
: coronary CTA
CK
: creatine kinase
LAD
: left anterior descending artery
LITA
: left internal thoracic artery
LMT
: left main trunk
MICS-CABG
: minimally invasive cardiac surgery coronary artery bypass grafting
SCAD
: spontaneous coronary artery dissection

## INTRODUCTION

The efficacy of CABG for SCAD remains controversial, as graft occlusion due to healing of SCAD lesions has been reported.^1)^ Since approximately half of the grafts were found to be occluded at the time of re-evaluation, the purpose of CABG is to ensure coronary blood flow in the acute phase. Published evidence on CABG for SCAD is limited, and the optimal surgical strategy for SCAD has not yet been established. We herein report a case of SCAD treated with MICS-CABG. We believe that this is the first reported case of SCAD treated with MICS-CABG in the literature.

## CASE PRESENTATION

**Fig. 1:**
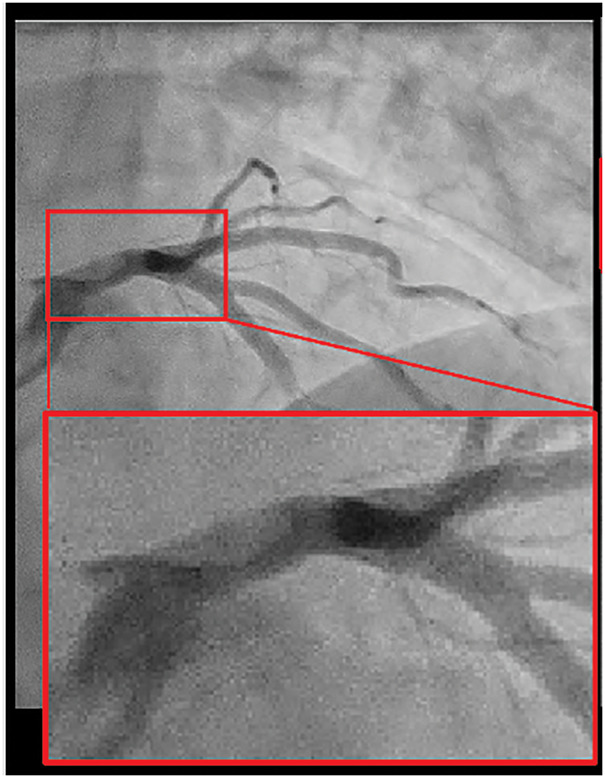
CAG on admission. CAG revealed double-lumen findings of the LMT and stenosis of the true lumen of the LMT. CAG, coronary angiography; LMT, left main trunk

**Fig. 2:**
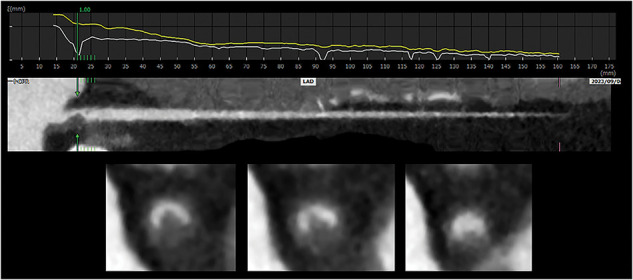
CCTA before surgery. CCTA showed 75% stenosis of the true lumen of the LMT due to compression by the false lumen. CCTA, coronary CTA; LAD, left anterior descending artery; LMT, left main trunk

**Fig. 3:**
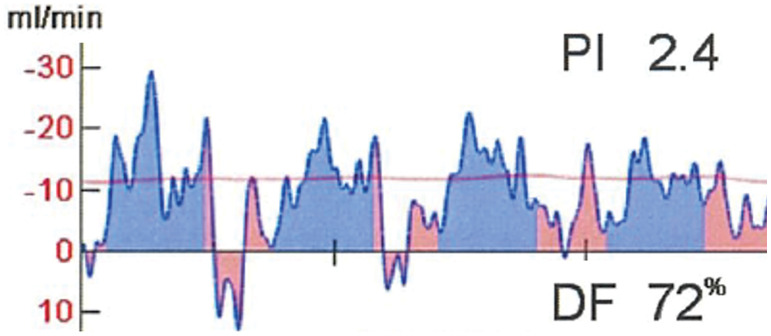
The image of the TTFM blood flow pattern. The graft blood flow rate was 20 mL/min, with a PI of 2.4 and a DF value of 72%. DF, diastolic filling; PI, pulsatile index; TTFM, transit-time flow measurement

A 50-year-old man was admitted to the emergency room of Toyota Memorial Hospital with the sudden onset of chest pain after exercise. He had no relevant medical history, but was a current smoker. His vital signs were stable, and his physical examination findings were unremarkable. A laboratory investigation showed a high troponin I level (178 pg/mL) and slightly elevated cardiac enzymes (AST 51 U/L, CK 258U/L). Electrocardiography revealed no ST elevation. Chest radiography revealed a cardiothoracic ratio of 49% without pleural effusion. ACS was suspected, and emergent CAG was performed. CAG revealed double-lumen findings in the LMT (**Fig. 1**). We diagnosed him with SCAD and decided to treat him conservatively according to the AHA guidelines, because his vital signs were stable and his chest pain disappeared. Despite medical treatment with heparin and nitrate, his troponin I (5549 pg/mL) and CK (533 U/L) levels increased the next day. CCTA revealed that the true lumen of the LMT was compressed to 75% stenosis by the false lumen (**Fig. 2**). While his vital signs remained stable, he was diagnosed with impending myocardial infarction that required coronary revascularization. We performed emergency CABG because percutaneous coronary intervention of the dissected LMT lesion was technically difficult. We hypothesized that coronary blood flow, including to the left circumflex region, could be maintained by bypass grafting only to the LAD. We therefore decided to perform MICS-CABG, which allows off-pump anastomosis of the LITA to the LAD without heart rotation. An 8-cm incision was made in the left anterior chest, just above the planned LAD anastomosis site. The LITA was harvested via the fifth intercostal space and anastomosed to the LAD under direct vision. The operation time was 182 min, and intraoperative blood loss was 368 mL. The graft blood flow rate was 20 mL/min, with a pulsatility index of 2.4 and a diastolic filling value of 72% (**Fig. 3**).

**Fig. 4:**
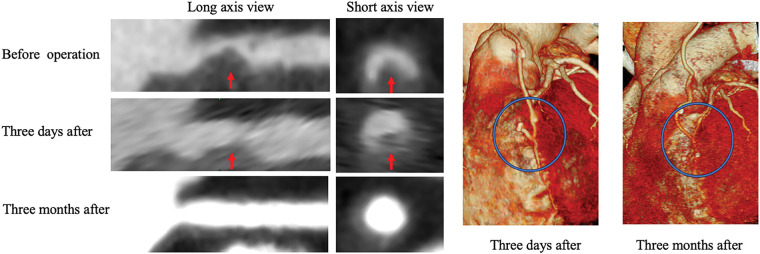
CCTA before and after the operation. Oppression of the true lumen (red arrows) gradually improved from 75% to approximately 50% at 3 days after bypass and completely disappeared at 3 months after surgery. Blue circles show LITA-LAD anastomosis site. CCTA, coronary CTA; LAD, left anterior descending artery; LITA, left internal thoracic artery

The postoperative course was uneventful. Postoperative bleeding was 35 mL/day and blood transfusion was not required. CCTA at 3 days and 3 months after the surgery revealed a patent LITA graft and improved LMT stenosis. Oppression of the true lumen gradually improved by approximately 50% at 3 days after CABG and completely disappeared 3 months after surgery (**Fig. 4**). The patient was discharged 1 week after surgery and returned to work 1 month after discharge.

## DISCUSSION

The true prevalence of SCAD remains uncertain, primarily because it is underdiagnosed. SCAD is still considered a rare disease that may cause 0.24% of ACS cases. It mainly occurs among working-age generations (mean age: 48.6 years for males; 52.3 years for females)^2)^ and is often associated with pregnancy or connective tissue abnormalities.

The optimal management strategy for SCAD has yet to be established. Conservative therapy is generally preferred in patients who are clinically stable, and such cases have generally been associated with favorable outcomes.^3)^ Hayes et al. demonstrated angiographic resolution of SCAD lesions in the majority of patients (70%–97%).^2)^ Nevertheless, recurrent myocardial infarction occurs in 5%–10% of conservatively managed patients, mainly due to extension of the dissection within the first week after the acute episode.^2,4)^

In contrast, published evidence on CABG for SCAD is limited, and its efficacy remains controversial. Several papers have reported late graft occlusion due to the healing of SCAD lesions. We have reported that 14 of the 24 grafts for SCAD (58%) were occluded on restudy.^1)^ Therefore, the purpose of CABG is to ensure coronary blood flow during the acute phase, and invasiveness should be considered when selecting the surgical strategy. In MICS-CABG, the left mini-thoracotomy approach avoids the need for a median sternotomy.

Although the rates of operative mortality and major morbidity are similar between conventional CABG and MICS-CABG, MICS-CABG is associated with fewer blood transfusions, shorter hospital stays, and faster return to full physical activity.^5)^ Since SCAD frequently occurs in pregnant women or individuals of working age, a surgical strategy that facilitates an earlier return to work represents a significant advantage. Previous reports have shown that the median time to recovery of physical activity is significantly shorter after MICS-CABG (12 days) than after off-pump CABG via median sternotomy (36 days).^6)^ Furthermore, because some patients with SCAD have underlying connective tissue abnormalities and may require future cardiac surgery, MICS-CABG offers the additional advantage of preserving the sternum and facilitating reoperation.

The disadvantages of MICS-CABG include difficulty harvesting the ITA graft and a narrow working space for coronary anastomosis. However, the advantages of MICS-CABG outweigh these disadvantages, and we believe that MICS-CABG is a useful alternative surgical strategy for SCAD, especially under relatively stable hemodynamic conditions. Moreover, extracorporeal membrane oxygenation support can be applied in cases of hemodynamic instability, even with MICS-CABG. In particular, LAD anastomosis through the left mini-thoracotomy approach does not require cardiac rotation, allows stable hemodynamics, and can be performed under off-pump. The LAD is the most commonly involved vessel (32%–46% of cases) in SCAD. By coronary territory, involvement of the LAD—including its diagonal and septal branches—accounts for 45%–61% of cases, whereas the left main artery is affected in up to 4%.^2)^

In MICS-CABG, the use of *in situ* grafts is preferred because proximal anastomosis to the ascending aorta is technically challenging. In CABG for SCAD, graft patency rates have been reported to be 55% for arterial grafts and 68.8% for venous grafts.^3)^ Although the patency of arterial grafts is inferior to that of saphenous vein grafts, arterial grafts can still provide sufficient flow support in the acute phase, and performing LITA-LAD anastomosis in MICS-CABG is an acceptable strategy.

## CONCLUSIONS

In patients with a relatively stable hemodynamic status, MICS-CABG is a feasible and effective therapeutic approach for SCAD.
